# Correlation between *MDM2* T309G single nucleotide polymorphism and esophageal cancer susceptibility: An updated meta‐analysis

**DOI:** 10.1111/1759-7714.13316

**Published:** 2020-01-22

**Authors:** Lele Yin, Guo Shen, Bin Zhu

**Affiliations:** ^1^ Department of Emergency Huaihe Hospital of Henan University Kaifeng China; ^2^ Department of General Surgery Zhuji Affiliated Hospital of Shaoxing University Shaoxing China; ^3^ Department of Emergency Lishui People's Hospital Lishui China

**Keywords:** Esophageal cancer, *MDM2* gene, meta‐analysis, polymorphism, susceptibility

## Abstract

**Background:**

The aim of this study was to investigate the correlation between *MDM2* T309G single nucleotide polymorphism (SNP) and esophageal cancer susceptibility through pooling the open published data.

**Methods:**

By systematic searching the databases of Medline, EMBASE, CBM and CNKI, the case‐control or cohort studies related to *MDM2* T309G single nucleotide polymorphism and esophageal cancer risk were screened. Genetic phenotype data of T309G single nucleotide was extracted from the original included studies. The correlation between *MDM2* T309G single nucleotide polymorphism and esophageal cancer susceptibility was demonstrated by the odds ratio (OR) and its corresponding 95% confidence interval (95% CI). Publication bias was investigated by Egger's line regression test and begg's funnel plot.

**Results:**

After systematic searching of the relevant database, nine publications were finally included in the present study. The combined data demonstrated that the subjects with the G genotype had an increased risk of developing esophageal cancer in dominant (OR = 1.13, 95% CI: 1.00–1.27, *P* = 0.043), recessive (OR = 1.27, 95% CI: 1.12–1.45, *P* = 0.000) and homozygous (OR = 1.34, 95% CI:1.04–1.74, *P* = 0.024) genetic model through random or fixed data pooling method. Both begg's and Egger's line regression test indicated no significant publication bias.

**Conclusion:**

Based on the present data, there was a significant correlation between *MDM2* T309G single nucleotide polymorphism and esophageal cancer susceptibility. Individuals with G genotype may have an increased risk of developing esophageal cancer.

## Introduction

Esophageal cancer is one of the most diagnosed malignant carcinoma of the digestive system. According to the latest statistical data of GLOBOCAN in 2018,^1^ there were approximately 570 000 new cases of esophageal cancer and 500 000 deaths, ranking the eighth incidence and seventh of all the malignant carcinomas. In year 2018, there were approximately 307 000 new cases of esophageal cancer and 283 000 deaths in China, ranking fifth in the incidence of malignant tumors and fourth in the mortality rate.^2^ As is already known, the development of esophageal cancer is the result of the interaction of genes and environmental factors, but the specific pathogenesis of both have not as yet been fully elucidated, and need further exploration.^3^ In recent years, studies have confirmed that gene single nucleotide polymorphism (SNP) was closely correlated with the cancer susceptibility.^4^

Murine double minute 2 (*MDM2*) locating in chromosome 12q15 encodes a nuclear‐localized E3 ubiquitin ligase. The encoded protein can promote tumor formation by targeting tumor suppressor proteins, such as p53, for proteasomal degradation. 309 T > G of *MDM2* gene is a common SNP site for human beings and considered to correlate with cancer susceptibility. Chen and colleagues^5^ discussed the 309 T > G SNP and esophageal cancer risk and published their meta‐analysis in 2015. In that study,^5^ the author only included six studies. Since five years have now past, several new studies have been published which are relevant to *MDM2* T309G single nucleotide polymorphism and esophageal cancer susceptibility. Here, we provide an updated meta‐analysis relevant to *MDM2* T309G single nucleotide polymorphism and esophageal cancer susceptibility by adding new publications.

## Methods

### Electronic searching of databases

A systematic search of the electronic databases of Medline, EMBASE, CBM and CNKI was performed using the following subject terms: *MDM2*, murine double minute 2, esophageal, esophagus, carcinoma or cancer or malignancy or neoplasm or tumor or tumor, all related names to the specified SNP: rs2279744 or SNP309, or T309G by two reviewers (L.L. Yin & G. Shen), respectively. The publication screening procedure was performed according to Cochrane's handbook. The screening results were also cross‐checked by the two aforementioned reviewers. The references of the studies included were also carefully screened in order to identify potentially suitable publications.

### Inclusion and exclusion criteria of studies

The publication inclusion criteria were as follows: (i) Case‐control or cohort studies relevant to *MDM2* T309G single nucleotide polymorphism and esophageal cancer susceptibility; (ii) papers were published in English or Chinese; (iii) the cases were patients diagnosed with esophageal cancer by pathology or cytology; and (iv) genotyping was correct. Publication exclusion criteria was as follows: (i) Case report or literature review publications relevant to *MDM2* T309G single nucleotide polymorphism and esophageal cancer susceptibility; (ii) studies published in other languages; (iii) duplicated publishing data; (iv) esophageal cancer not confirmed by pathology or cytology; and (v) the genotype of GG, TG and TT could not be directly extracted or calculated from the original studies.

### Data extraction

The general information and genotyping data of each individual study was individually extracted by two reviewers (G. Shen and B. Zhu). The main information such as first author, journal, control type and Hardy‐Weinberg equilibrium of the control group were extracted from the original included studies. The genotype of *MDM2* T309G distribution were also extracted and cross checked.

## Statistical analysis

Stata11.0SE was applied for data analysis. The correlation between *MDM2* T309G single nucleotide polymorphism and esophageal cancer susceptibility was expressed by the odds ratio (OR) and 95% CI. The statistical heterogeneity was assessed by I^2^ test. The OR was combined through the random or fixed effect method. The publication bias was evaluated by begg's funnel plot and Egger's line regression test.

## Results

### Main characteristics of studies included

**Figure 1:**
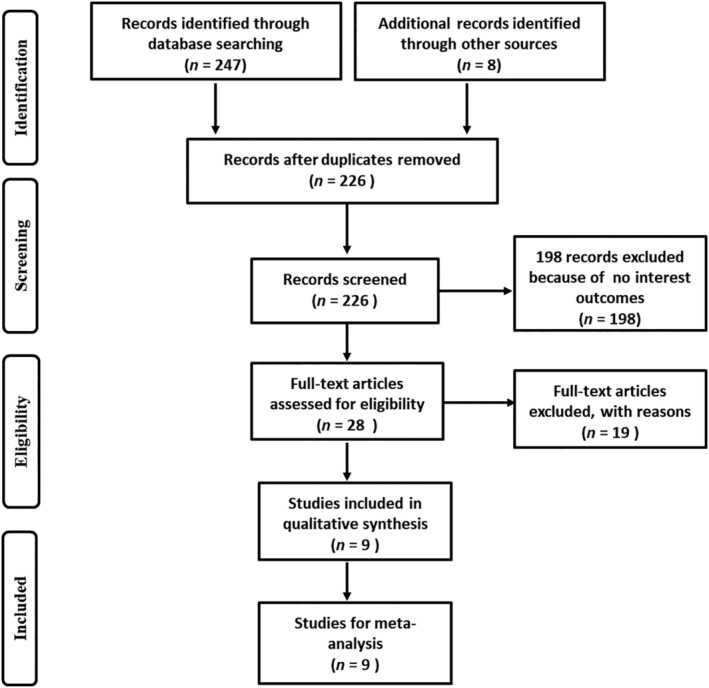
The publication electronic searching flow chart.

Nine studies^6^, ^7^, ^8^, ^9^, ^10^, ^11^, ^12^, ^13^, ^14^ relevant to *MDM2* T309G single nucleotide polymorphism and esophageal cancer susceptibility fulfilled the inclusion criteria and were included in the meta‐analysis (Fig 1). Of the nine publications included, eight patients were of Asian origin and one of Caucasian origin. Five studies used population based healthy controls and the other four used hospital‐based controls. The main characteristics of the nine studies included are showed in Table 1.

**Table 1:** Main information of the nine original studies

				Case			Control			
Author	Year	Ethnicity	Control type	TT	TG	GG	TT	TG	GG	Hardy‐Weinberg equilibrium
Hong *et al*.	2005	Asian	Population based	203	348	207	418	711	291	Yes
Cao *et al*.	2007	Asian	Hospital based	50	170	131	117	299	226	Yes
Liu *et al*.	2010	Caucasian	Population based	116	154	41	175	199	80	Yes
Ma *et al*.	2012	Asian	Population based	49	119	58	50	118	58	Yes
Er *et al*.	2012	Asian	Hospital based	47	31	43	41	78	23	Yes
Yang *et al*.	2013	Asian	Population based	163	126	18	161	126	24	Yes
Zhang *et al*.	2015	Asian	Population based	37	70	25	47	71	14	Yes
Er *et al*.	2009	Asian	Hospital based	23	51	32	39	46	21	Yes
Li *et al*.	2011	Asian	Hospital based	37	70	25	47	71	14	Yes

### TG and GG genotype distribution

**Figure 2:**
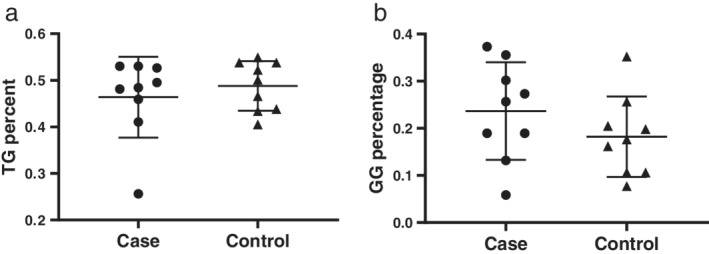
Scatter plot of genotype distribution in esophageal cancer and control group. (**a**) TG genotype distribution between esophageal cancer and healthy controls; (**b**) GG genotype distribution between esophageal cancer and healthy controls.

Before pooling the data, we first calculated the frequency of the TG and GG genotypes. The median TG and GG genotype frequency were 0.4843 and 0.2566 in the esophageal cancer group. For the control group, the median TG and GG genotype frequency were 0.5007 and 0.1762 (Fig 2).

### Statistical heterogeneity

Statistical heterogeneity of each genetic model was assessed using the I^2^ test. For the dominant genetic model (GG + TG vs. TT), the statistical heterogeneity was not statistically different with the I^2^ = 36.6%, *P* = 0.125; However, for the recessive (GG vs. TT + TG, I^2^ = 69.9%, *P* = 0.001) and homozygous genetic models (GG vs. TT, I^2^ = 53.8%, *P* = 0.027), the statistical heterogeneity was statistically significant. Therefore, data was pooled through the fixed effect method in the dominant genetic model and the random effect method in the recessive and homozygous genetic models, respectively.

### Data combination in dominant genetic model (TG + GG vs*.* TT)

**Figure 3:**
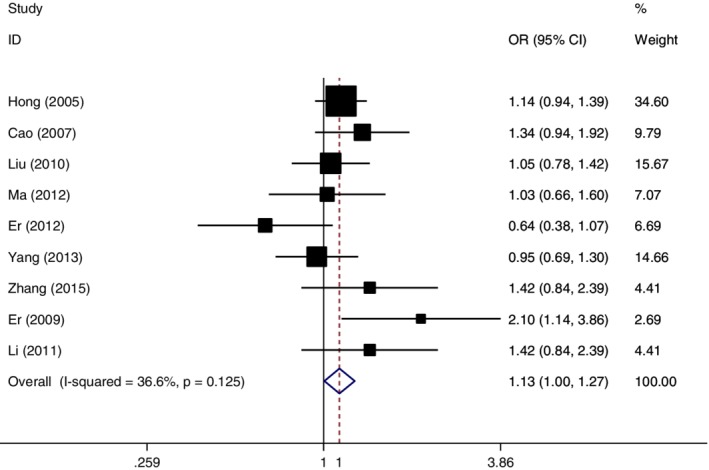
Forest plot of OR in evaluation of the *MDM2* T309G single nucleotide polymorphism and esophageal cancer susceptibility through the fixed effect method in the dominant genetic model.

Without statistical heterogeneity, the odds ratio for *MDM2* T309G single nucleotide polymorphism and esophageal cancer susceptibility was pooled by the fixed effect model. The combined OR = 1.13 (95% CI: 1.00–1.27, *P* = 0.043), which indicated subjects with TG or GG genotype had increased risk of developing esophageal cancer in the dominant genetic model (Fig 3).

### Data combination in recessive genetic model (GG vs. TT + TG)

**Figure 4:**
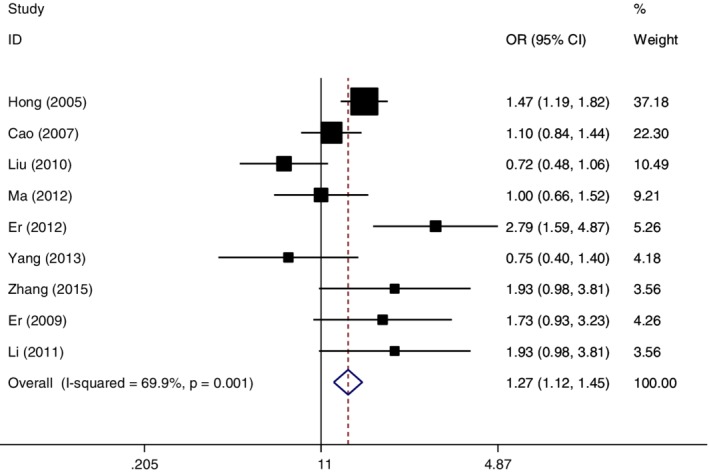
Forest plot of OR in evaluation of the *MDM2* T309G single nucleotide polymorphism and esophageal cancer susceptibility through the random effect method in the recessive genetic model.

In the recessive genetic model, the data was pooled by the random effect method. The combined OR = 1.27 (95% CI: 1.12–1.45, *P* = 0.000), which demonstrated the subjects with GG genotype were more susceptible to esophageal cancer compared with the TT or TG genotype in the recessive genetic model (Fig 4).

### Data combination in homozygous genetic model (GG vs. TT)

**Figure 5:**
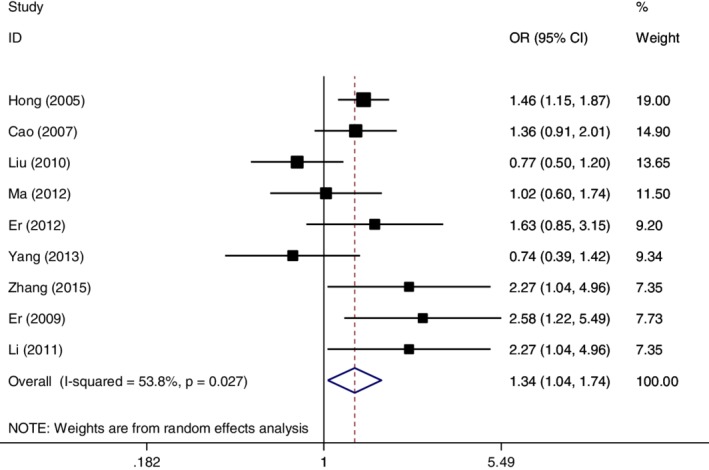
Forest plot of OR in evaluation of the *MDM2* T309G single nucleotide polymorphism and esophageal cancer susceptibility through the random effect method in the homozygous genetic model.

With regard to the homozygous genetic model (GG vs. TT), the OR was combined by the random effect method because of statistical heterogeneity across the nine original publications. The pooled OR = 1.34 (95% CI:1.04–1.74, *P* = 0.024), which indicated subjects with GG genotype had an increased risk of developing esophageal cancer in the homozygous genetic model (Fig 5).

### Publications bias evaluation

**Figure 6:**
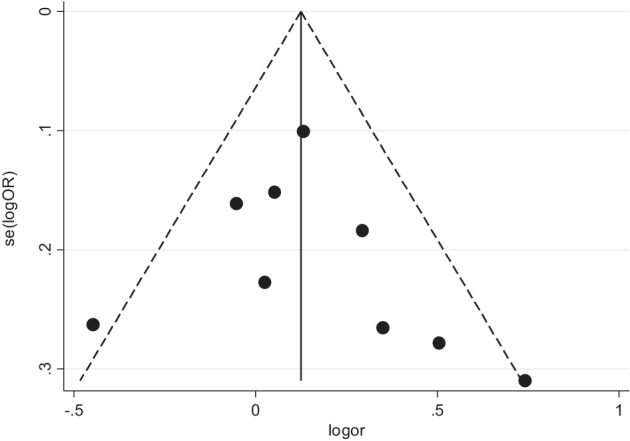
Begg's funnel plot was used to investigate publication bias in the dominant genetic model (GG + TG vs. TT).

**Figure 7:**
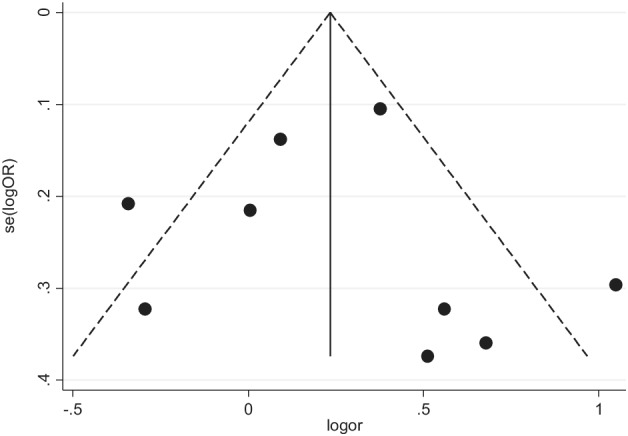
Begg's funnel plot was used to investigate publication bias in the recessive genetic model (GG vs. TT + TG).

**Figure 8:**
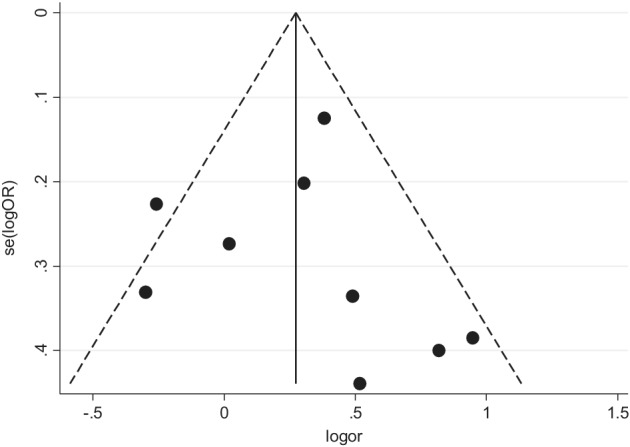
Begg's funnel plot was used to investigate publication bias in the homozygous genetic model (GG vs. TT).

The publication bias of the aforementioned three genotypes was assessed through begg's funnel plot and Egger's line regression test. The begg's funnel plot was generally left‐right symmetrical in the dominant (Fig 6), recessive (Fig 7) and homozygous (Fig 8) genetic model. The Egger's line regression test also indicated no significant publication bias (Table 2).

**Table 2:** Egger's line regression test for evaluation the publication bias

Genetic model	Coefficient	SE	*t* value	*P*‐value	95% CI
Dominant	0.668	1.228	0.54	0.603	−2.23 to 3.57
Recessive	0.499	1.547	0.32	0.756	−3.15 to 3.55
Homozygous	0.485	1.295	0.37	0.719	−2.58 to3.55

## Discussion

In the present updated meta‐analysis, nine case‐control studies relevant to *MDM2* T309G single nucleotide polymorphism and esophageal cancer susceptibility were included. There were eight studies on patients of Asian origin and only one publication on a patient of Caucasian origin. The pooled data showed there was a significant correlation between *MDM2* T309G single nucleotide polymorphism and esophageal cancer susceptibility. This indicated that subjects with the G genotype had an increased risk of developing esophageal cancer in dominant (OR = 1.13, *P* = 0.043), recessive (OR = 1.27, *P* = 0.000) and homozygous (OR = 1.34, *P* = 0.024) genetic models through the random or fixed method. Chen and colleagues discussed the 309 T > G SNP and esophageal cancer risk by meta‐analysis in year 2015 and found that *MDM2* T309G SNP was correlated with esophageal cancer susceptibility. Compared with the previously published meta‐analysis performed by Chen *et al*. our study added three new publications with increased statistical power and achieved the same conclusion.

*MDM2* is itself transcriptionally‐regulated by p53.^15^ Overexpression or amplification of this gene has been detected in a variety of malignant carcinomas.^16^, ^17^ Studies have also determined that *MDM2* 309 T > G SNP were also correlated with an increased risk of solid tumors. Luan *et al*. reported that *MDM2* T309 G polymorphism may contribute to NSCLC susceptibility, especially in the Asian population and women.^18^ Li *et al*. found that the GG genotype of *MDM2* SNP309 was significantly associated with an increased endometrial cancer risk by the meta‐analysis.^19^ In our meta‐analysis, we also confirmed the G allele could increase the esophageal cancer susceptibility, which was in accordance with previous publications. However, the exact mechanism of how *MDM2* T309 G SNP affects cancer susceptibility has not yet been fully elucidated. Knappskog and colleagues found that *MDM2* T309 G SNP affected cancer risk through modulation of Sp1 transcription factor binding.^20^ Other researchers reported that key SNP changes of *MDM2* may have a large impact on the activity of p53‐dependent tumor suppression.

Although the exact pathogenesis of how SNP changes *MDM2* and cancer susceptibility are not fully understood, a significant correlation between *MDM2* T309 G SNP and esophageal cancer has been confirmed by our present meta‐analysis.

However, the present study has several limitations that need to be considered. First, only studies published in English or Chinese were searched and included in the meta‐analysis. This may have limited the number of potential articles retrieved. Second, only three new studies have been added to the work compared to the previous meta‐analysis. Third, due to the significant heterogeneity across the included studies, the statistical power was limited.

## Disclosure

The authors confirm that there are no conflicts of interest.
